# Surgical delay increases the survival of expanded random-pattern flap in pediatric patients

**DOI:** 10.1038/s41598-023-45852-3

**Published:** 2023-11-06

**Authors:** Jeong Hyun Ha, Se Yeon Lee, Tae Hyun Choi, Seong Oh Park

**Affiliations:** 1 Department of Plastic and Reconstructive Surgery, Seoul National University Hospital, Seoul, Republic of Korea; 2 Interdisciplinary Program of Medical Informatics, Seoul National University College of Medicine, Seoul, Republic of Korea; 3 The Nevus Plastic Surgery Clinic, Seoul, Republic of Korea; 4 Department of Plastic and Reconstructive Surgery, Hanyang University College of Medicine, Seoul, Republic of Korea

**Keywords:** Skin diseases, Medical research, Paediatric research

## Abstract

Despite the aid of tissue expansion, the ideal design of random pattern flap is not always available in patients with extensive skin lesions. We investigated the effectiveness of surgical delay on expanded flaps in pediatric patients. Retrospective cohort study was performed on patients who underwent tissue expansion surgery for extensive skin lesions at Seoul National University Children’s Hospital. The surgical delay technique was employed for patients with unfavorable flap conditions related to location or transposition angles. The dimensions of skin lesions and flaps were measured based on medical photographs. Fifty patients underwent a total of 66 tissue expansion procedures (49 conventional procedures among 41 patients, 17 surgical delay procedures among 15 patients) from January 2016 to September 2019. Although flaps in the surgical delay group were more narrow-based (*p* < 0.001), the partial flap loss rate and excised area-to-inflation amount ratio was comparable between the two groups (*p* = 0.093 and *p* = 0.194, respectively). Viable flaps, excluding postoperative necrosis, in the surgical delay group were significantly more narrow-based in terms of the length-to-base width ratio and the area-to-base width ratio compared to conventional group (*p* < 0.01, *p* < 0.01). Surgical delay can result in outcomes comparable to well-designed random flaps, even in disadvantageous conditions. Patients with large skin lesions but limited areas for expansion may benefit from surgical delay.

## Introduction

Random pattern flaps may not always be ideally designed considering vascular territory. When adjacent normal tissue is limited compared to an extensive skin lesion, tissue expansion can generate tissue to reconstruct defects. Tissue expansion is widely used as a reconstructive option for extensive skin lesions, including giant nevi, alopecia, and burn scars. It increases the viable flap size and enables the primary closure of the donor site. However, extensive skin lesions reduce the area available for tissue expansion and flap elevation, particularly in pediatric patients^1^. Expander placement and flap design may not always be in the best conditions. Back-cuts, rotation flaps, or transposition flaps are used to maximize the use of the expanded tissue^2^. Random-pattern flaps after expansion are known to have greater survival compared to non-expanded tissues^3,4^. Nevertheless, in some circumstances, expanded flap may be at risk of poor vascularity related to location and size of the lesion. Despite the aid of tissue expansion, expanded flaps may not be maximally utilized in unfavorable conditions.

When the expander is inevitably placed in an unfavorable location, a long back-cut can narrow the flap base, compromising flap circulation. Excessive rotation, particularly in distant flaps for forearm lesions, can lead to kinking in the flap pedicle, posing a threat to vascularity^5^. Prior history of wound problems or severe scarring can also result in poor circulation, potentially causing necrosis at the flap’s distal tip. In such circumstances, surgical excision may be limited, requiring a greater number of operations for tissue expansion.

The surgical delay technique offers a valuable option for improving flap survival under unfavorable conditions, evolving from unpredictable to reliable. Surgical delay can be performed by making incisions or partially elevating and re-suturing the flap prior to the definite surgery^6,7^. In recent years, refined flap elevation techniques and advancements in surgical procedures have increased flap survival rates, reducing the necessity of delay procedures.

Nevertheless, selective random pattern flaps, such as flaps unavailable for anatomical elevation, displaying poor vascularity, or in pediatric patients with limited donor sites, can still benefit from the delayed technique^8^. To our knowledge, there is no clinical report combining the effects of tissue expansion and the delayed phenomenon in soft tissue reconstruction. Our study aimed to investigate the effectiveness and usefulness of the surgical delay technique following tissue expansion in pediatric patients and evaluate whether surgical delay could improve flap survival to some extent under adverse conditions.

## Methods

We conducted a retrospective cohort study at Seoul National University Children’s Hospital, focusing on patients who underwent tissue expander insertion surgery for extensive skin lesion between January 2016 and September 2019. Extensive skin lesions included giant nevi, burn scars, and alopecia.

We excluded patients who used two or more expanders simultaneously in adjacent areas and those whose flaps were divided and transposed to different sites. Conventional tissue expansion typically involved a two-stage procedure. In the first stage, a tissue expander is inserted at a location free from the lesion. Tissue expansion is carried out over an interval of of three to 6 months with weekly inflations. After the final tissue expansion, the expander is removed along with excision of the skin lesion, and the expanded flap is utilized for defect coverage as part of the second-stage operation.

In our study, we introduced the surgical delay procedure, which was performed before the expander removal operation. This additional step was considered in specific conditions: (1) when the flap was suspected to have poor vascularity, or (2) when the long axis of the skin lesion was aligned with the axis for flap transfer, necessitating true advancement flap for defect coverage. Poor flap perfusion was suspected when the flap size was notably larger than its base or when a random pattern flap was considered larger than the required angiosome.

We initially reviewed all patients who underwent surgical delay and compared their characteristics and outcomes with those of patients who did not. The study was after conducted with the approval of the Seoul National University Hospital Institutional Review Board (IRB No. H-1805-094-946) with a waiver of informed consent granted by the IRB. All methods were carried out in accordance with relevant guidelines and regulations.

### Operation technique

An expander was inserted in the plane of subcutaneous fat in face, subgaleal layer in scalp, or suprafascial layer in other regions. The size of expander was determined considering the size of the lesion and the area of normal adjacent skin. Inflation was started from 3 weeks post-operation on a weekly basis and was done as much as possible, but not causing skin pallor. When a flap was suspected to be at risk of partial necrosis according to above indications, delay procedure was performed one or two times, within 3–14 days prior to expander removal. The delay procedure was performed by making an incision along the borders of the planned flap territory, covering up to 70%. The incision was made down to the partial subcutaneous layer to prevent expander exposure, and skin key sutures were used to prevent contraction. In cases where a back-cut was anticipated, it was not included in the initial incision for the delay procedure but was made during the final surgery in all cases when necessary.

### Evaluation

Perioperative photographs and follow-up photographs were evaluated for characteristics of excised skin lesions and local flaps. Image J software was used to measure the surface area of the excised skin lesion as well as the length and the width of the flap. To account for the effect of shrinkage after excision, we calculated the ratio of change in surface area with three random samples of excised lesion, which was 0.757. We first thoroughly reviewed patients who underwent surgical delay. Then we compared flap survival between cases with and without surgical delay (Surgical delay group and conventional group, respectively). Ratios of flap length-to-base width and of flap area-to-base width were calculated in elevated flaps during surgery and viable flaps after surgery. These ratios were compared between groups to evaluate the efficacy of the surgical delay procedure in enhancing the vascularity of random pattern flaps.

### Statistical analysis

Patient demographics were compared between the conventional group and the surgical delay group. Statistical analyses of 2 × 2 contingency tables of categorical variables were performed using the Fisher’s exact test. For continuous variables, we performed the Mann–Whitney test for comparisons. All statistical tests were two-sided and significance was defined as *p* < 0.05. All analyses were performed using the Statistical Package for the Social Sciences for Windows Version 26.0 (IBM, Chicago, IL, USA).

## Results

A total of 55 patients underwent 66 tissue expansion procedures. Demographic data are summarized in Table 1. The average age of patients at tissue expander insertion was 52.5 months (range 12.0–235.0). The conventional group included 49 tissue expansion procedures whereas the surgical delay group included 17 procedures. Nine patients underwent twice of tissue expansions, including conventional procedure at first and then with surgical delay. Two patients underwent tissue expansions three times, including one conventional procedure then twice of surgical delays. The analysis of flap characteristics revealed that distant flaps were performed in seven cases in the surgical delay group and two cases in the conventional group.

**Table 1:** Patient characteristics.

Patient demographics	Surgical delay group (n = 17)	Conventional group (n = 49)	Total
Age (years, range)	6.1 (1.0–17.6)	6.2 (1.0–19.6)	
Sex			
Male	7	22	29
Female	10	27	37
Diagnosis			
Giant nevus	16	49	65
Brun scar	1	0	1
Location of lesion			
Upper extremity	7	4	11
Lower extremity	3	3	6
Scalp	1	7	8
Face	4	11	15
Trunk	2	24	26
Distant flap	7	2	9
Previous expansion*	6	5	10

Surgical characteristics			p-value
Expander volume (cc)	220.3 (45–400)	205.0 (45–560)	0.673
Total inflation amount (cc)	444.3 (119–901)	446.0 (72–1040)	0.764
Area of excised lesion (cm^2^)	76.7 (21.5–117.5)	82.1 (12.4–176.7)	0.505
Excised area-to-inflation ratio	0.18 ± 0.07	0.22 ± 0.09	0.194
Elevated flap			
Length-to-base width ratio	2.45 ± 0.62	1.32 ± 0.41	< 0.01
Area-to-base width ratio	13.63 ± 5.55	10.28 ± 4.66	0.25
Survived flap			
Length-to-base width ratio	2.38 ± 0.59	1.32 ± 0.40	< 0.01
Area-to-base width ratio	13.37 ± 5.54	10.23 ± 4.59	< 0.01
Partial flap loss (%)	3/17 (17.6%)	2/49 (4.1%)	0.103
Wound revision	1	0	
Secondary healing	2	2	

*Patients who has history of tissue expansion at the same location.

### Effectiveness of surgical delay technique

Flaps in the surgical delay group were significantly narrow-based with a higher length-to-base width ratio (*p* < 0.001), indicating unfavorable condition for flap survival. However, the partial flap loss rate did not differ significantly (*p* = 0.093), and the excised area-to-inflation amount ratio was comparable between the two groups (*p* = 0.194). When evaluating the area of viable flaps, excluding the postoperative necrotic area, viable flaps in the surgical delay group were significantly narrow-based with a higher length-to-base width ratio and area-to-base width ratio (*p* < 0.01, *p* < 0.01). Flap perfusion was well maintained even with a narrow base, with an aid of surgical delay. Distal flap necrosis occurred in 3/17 cases in the surgical delay group and 2/49 cases in the conventional group (*p* = 0.103). Among the three cases in the surgical delay group developing distal flap necrosis, one required surgical revision, whereas the other two were healed with secondary intention. (Table 1).

### Details of the surgical delay group

A total of 17 cases underwent surgical delay before expander removal and local flap procedure. The location of the skin lesion was the extremity in 10 cases, the back in 2 cases, and the head and neck region in 5 cases. In cases with extremity lesions, the expander was placed in an adjacent area in 3 lower extremity cases and at the trunk in 7 upper extremity cases for distant flaps. All patients in the surgical delay group were at high risk of flap necrosis due to various unfavorable conditions. Six cases had a history of tissue expansion at the same location, and four of them required wound revision after tissue expander insertion. Three cases underwent expander changes due to suspected leakage or surgical site infection. The angle of flap transposition was over 90 degrees in 6 cases, suggesting the risk of pedicle kinking. In 2 cases, the nevus lay in the same axis of the flap advancement, which was unfavorable for nevus removal. The average length-to-width ratio was 2.45 (SD 0.62) in elevated flaps and 2.38 (SD 0.59) in survived flaps, respectively. The average area-to-width ratio was 13.63 (SD 5.55) in elevated flaps and 13.37 (SD 5.54) in survived flaps.

**Figure 1:**
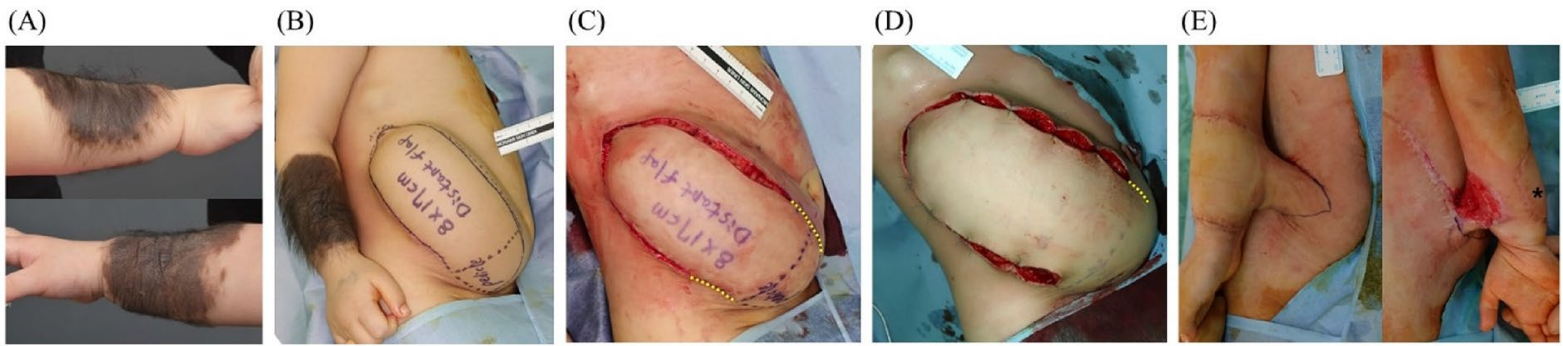
A 23-months-old female patient underwent tissue expansion on her abdomen for the reconstruction of her Rt. Forearm nevus lesion (**A**). 8 × 17 cm narrow based distant flap was designed (**B**). Surgical delay procedures (1st and 2nd) were performed to enhance flap circulation (**C**, **D**). The flap was successfully transferred without necrosis and detached from the abdomen (**E**). (Yellow dotted line is marked along planned flap border; * is marked at distal tip of the flap).

**Figure 2:**
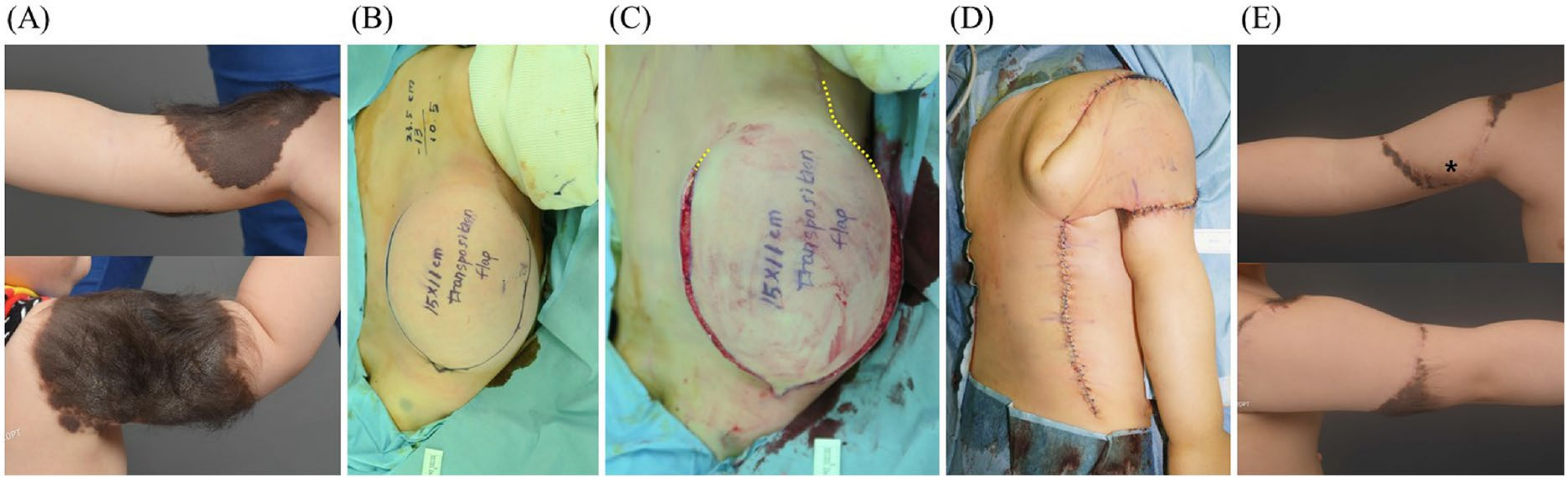
A 17-month-old male patient underwent tissue expansion on his back for the reconstruction of his Rt. Upper extremity (**A**). An 11 × 15 cm distant flap with a more than 90-degree angle of transposition, carrying a high risk of pedicle kinking, was designed (**B**). A surgical delay procedure was conducted to improve flap circulation (**C**). The flap was transferred to his Rt. upper extremity (**D**), resulting in successful reconstruction without flap necrosis (**E**). (Yellow dotted line is marked along planned flap border; * is marked at distal tip of the flap).

**Figure 3:**
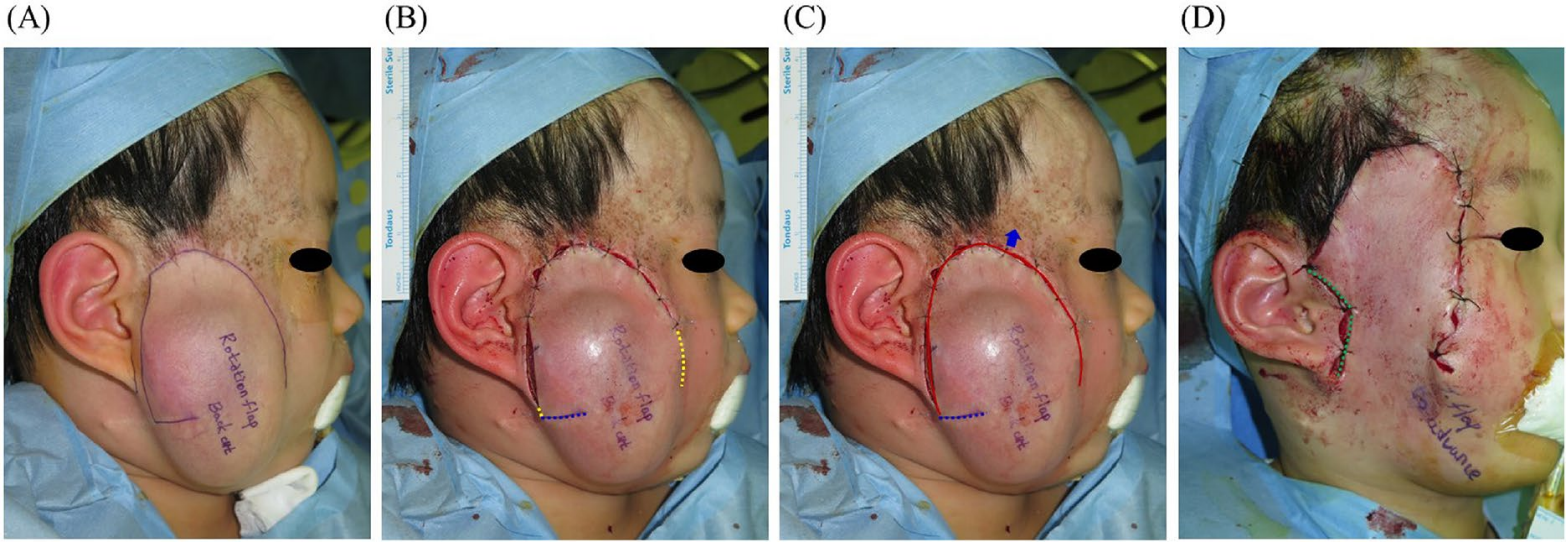
A 52-month-old male patient underwent tissue expansion on his cheek for facial reconstruction. A flap with an anticipated back-cut was designed (**A**). A surgical delay procedure was performed to enhance flap circulation, but the back-cut was not incised (**B**). During the final surgery, the angle and length of the back-cut were determined to transfer the flap most effectively (**C**, **D**). (Yellow dotted line is marked along planned flap border; Blu dotted line is marked along the anticipated back-cut; Green dotted line is marked along the actual back-cut made).

Three cases developed distal flap necrosis. Possible reasons for the tip necrosis include kinking of the pedicle or poor vascularity of the wound bed. In the first case, the flap was twisted three-dimensionally for distant flap coverage and the pedicle was rotated with tension. The flap had a length-to-width ratio of 2.6 and an area-to-width ratio of 14.6, which may have contributed to tip circulation deterioration despite the delay procedure. Two instances of distal flap necrosis occurred in one patient with a giant hairy nevus on the left leg. This patient had a history of six serial excisions at another clinic, with expander changes due to wound dehiscence and leakage. Circulation was likely compromised due to multiple operation and scarring. Despite performing surgical delay before the local flap, necrosis occurred at the distal tip. Further demographic details can be found in Table 2, and patient photographs are shown in Figs. 1, 2 and 3.

**Table 2:** Details of surgical delay cases.

No	Sex	Age (years)	Location	Flap transposition angle	Flap length-to-width ratio	Flap area-to-width ratio	Survived flap area-to-width ratio	Survived flap length-to-width ratio	Number of Surgical Delay	Complication	Delay interval (days)	Others
1	F	3.0	Cheek	< 90	3.1	9.2	9.2	3.1	1	0	6	Re-expansion
2	F	8.2	Back	< 90	2.5	5.7	5.7	2.5	2	0	7	Re-expansion
3	M	7.0	U/E	> 90	2.6	14.6	12.4	2.0	2	Distal flap necrosis	7	Distant flap
4–1	F	4.4	L/E	> 90	2.4	14.2	13.1	2.2	2	Distal flap necrosis	7	Re-expansion history of multiple laser therapy
4–2	F	2.7	L/E	> 90	3.5	14.1	13.0	3.1	1	Distal flap necrosis	2	Expander change (leakage) history of multiple laser therapy
5	F	1.5	U/E	> 90	2.8	19.8	19.8	2.8	2	0	7	Distant flap
6	M	1.0	U/E	> 90	2.2	26.9	26.9	2.2	2	0	7	Expander change (leakage, 2 times) distant flap
7	M	3.4	U/E	> 90	2.2	12.7	12.7	2.2	1	0	3	Expander change (leakage) distant flap
8	M	1.5	U/E	< 90	0.9	18.1	18.1	0.9	2	0	7	Expander change (infection) distant flap
9	M	5.5	Temple	< 90	2.2	6.6	6.6	2.2	2	0	6	Re-expansion
10	F	10.7	Back	< 90	1.8	17.0	17.0	1.8	1	0	4	Re-expansion
11	F	5.3	U/E	< 90	3.2	18.1	18.1	3.2	2	0	7	Distant flap
12–1	F	15.6	Face	> 90	2.6	12.2	12.2	2.6	2	0	14	
12–2	F	17.6	Face	< 90	2.4	8.8	8.8	2.4	2	0	7	Re-expansion
13	M	8.5	L/E	< 90	2.8	5.5	5.5	2.8	1	0	2	
14	F	3.2	U/E	< 90	2.8	13.6	13.6	2.8	1	0	5	Distant flap
15	M	3.5	Neck	< 90	1.7	14.6	14.6	1.7	2	0	6	

*U/E* upper extremity, *L/E* lower extremity. Delay intervals, time gap between first preconditioning and expander removal; Re-expansion, expander insertion at previously tissue expanded site;

All cases with expander change were performed after expander leakage.

Re-expansion cases correspond to cases which have history of previous tissue expansion.

## Discussion

Surgical delay is a procedure to enhance the vascularization of the flap^9^. The concept was introduced in early reports of delay procedure in nasal reconstruction or upper arm flaps. Since then, many animal experimental studies were reported for optimal time and method of the delay^6,10–13^. Myers and Cherry^14^ reported that the effect of delay began after 48 h and its maximal effect was at 8 days. On the other hand, Milton et al.^6^ concluded that the effect was maximum at about 2 weeks. The delayed phenomenon takes advantage of vasodilation and reorientation of choke vessels under ischemic condition to ensure the vascular supply in any desired flap^9,10^. The surgical delay resulting in opening up choke vessels between adjacent perforators, especially along the axis of the flap, has been established through experimental animal studies^10^. Sympathetic denervation, neovascularization, metabolic adaptation to ischemic conditions are also suggested mechanisms for delay procedure^15–19^.

Our delay procedure includes skin incision down to the fascia, partial flap elevation, or ligation of feeding vessel, causing partial devascularization of flap, prior to final transposition^12^. Soft tissue expansion inadvertently accompanies surgical delay during incision and undermining for expander insertion^10,20^. It results in opening choke vessels and vessel hypertrophy. We tried additional surgical delay procedure after expansion and before flap surgery to increase the viable flap length^10,19,20^. Our delay procedure included additional incision besides prior incision for expander insertion, which enables the reliable survival of expanded tissue flap even under unfavorable conditions. By utilizing this concept, we expected that delay procedure in soft tissue expansion can effectively remove the lesions even under unfavorable conditions by allowing choke vessels to dilate between adjacent perforators. We tried to apply this principle in pediatric patients undergoing soft tissue expansion, who have limited size of viable tissue for expansion, and evaluated its usability.

The surgical delay procedure itself can be a burden for pediatric patients because it requires additional surgical steps. However, when considering a series of process, including expander insertion, serial inflation, and expander removal, surgical delay may benefit patients in the long term by increasing the area of excision. Surgical delay is usually performed for several reasons, such as to expand the survival length of flaps, to enhance the vascularity in flaps of uncertain viability, and to improve the circulation of flaps to withstand the physical insults, including twisting or folding during transfer^13^. Indications for our surgical delay cases were similar to these mentioned conditions. Lesions requiring narrow-based flap, presumable poor vascularity after previous scarring or operation history, excessive flap transposition angle twisting the flap base, such as in some distant flap, were such situations. Extremity lesions lack appropriate location for expander placement are especially at risk of such circumstances. Upper extremity lesions are frequently covered by trunk flap after tissue expansion by distant flap^21,22^. However, that is not applicable for lower extremity lesions, which usually require expander placement in the same limb. In such cases, relatively narrow flaps with large rotation arcs are required for the defect coverage.

Surgical delay in patients with potentially poor vascularized flaps was successful, as indicated by a comparable area of lesion excised and a similar partial flap loss rate when compared to the conventional group (*p* = 0.103). This was achieved despite unfavorable flap conditions. Significantly, narrow based flap did not result in increase of flap loss when performed with surgical delay. Cherry et al.^3^ has reported that surviving lengths of random-pattern expanded flaps show 117% increase compared to acutely raised random-pattern flaps without expansion. Survival length of delayed flap is increased by 73% over that of acutely raised flap. Flap survival may be enhanced when combining tissue expansion with the delay procedure. Among 17 patients in our surgical delay group, distal flap necrosis occurred in patients who had three or more of following conditions: flap length-to-width ratio over 2.5, distant flap, extremity lesion, flap transposed angle over 90, or re-expansion of previously expanded site. Each condition may be considered as a risk factor for flap loss after soft tissue expansion. In cases with several risk factors, flap should be conservatively elevated with limited length, even when accompanied by delay procedure.

Our study nevertheless has several limitations. First, our study is a retrospective design with limited sample size. Effect of delay procedure could not be accurately analyzed because unfavorable flaps were not randomly allocated but were all included in the surgical delay group. Second, the evaluation and measurements were performed using photographs. Exact measurement would be possible if performed directly. Further prospective randomized control study with a larger sample size is needed to draw firm conclusion about the effect of surgical delay after tissue expansion. Direct perfusion assessment such as indocyanine green angiography after surgical delay prior to flap surgery may be helpful in accurately evaluating the effect of delay procedure and for safe and successful flap elevation. Our study was conducted in a pediatric population. However, the surgical delay technique could benefit not only pediatric patients but also individuals with extensive skin lesions that require removal and reconstruction.

In conclusion, surgical delay can result in comparable outcome to well-designed random flaps overcoming disadvantageous conditions. Surgical delay may be beneficial if used in selected patients, even though it involves extra steps of operation. It may benefit pediatric patients who have unfavorable conditions regarding locations or transposition angles.

## Data Availability

The data that support the findings of this study are available from the corresponding author upon reasonable request.
